# Reconstruction of the lower limb bones from digitised anatomical landmarks using statistical shape modelling

**DOI:** 10.1016/j.gaitpost.2020.02.010

**Published:** 2020-03

**Authors:** Daniel Nolte, Siu-Teing Ko, Anthony M.J. Bull, Angela E. Kedgley

**Affiliations:** Department of Bioengineering, Imperial College London, London, SW7 2AZ, United Kingdom

**Keywords:** Musculoskeletal modelling, Motion capture, Hip joint centre, Soft tissue artefact, Landmark digitisation, Statistical shape model

## Abstract

**Background:**

Bone shapes strongly influence force and moment predictions of kinematic and musculoskeletal models used in motion analysis. The precise determination of joint reference frames is essential for accurate predictions. Since clinical motion analysis typically does not include medical imaging, from which bone shapes may be obtained, scaling methods using reference subjects to create subject-specific bone geometries are widely used.

**Research question:**

This study investigated if lower limb bone shape predictions from skin-based measurements, utilising an underlying statistical shape model (SSM) that corrects for soft tissue artefacts in digitisation, can be used to improve conventional linear scaling methods of bone geometries.

**Methods:**

SSMs created from 35 healthy adult femurs and tibiae/fibulae were used to reconstruct bone shapes by minimising the distance between anatomical landmarks on the models and those digitised in the motion laboratory or on medical images. Soft tissue artefacts were quantified from magnetic resonance images and then used to predict distances between landmarks digitised on the skin surface and bone. Reconstruction results were compared to linearly scaled models by measuring root mean squared distances to segmented surfaces, calculating differences of commonly used anatomical measures and the errors in the prediction of the hip joint centre.

**Results:**

SSM reconstructed surface predictions from varying landmark sets from skin and bone landmarks were more accurate compared to linear scaling methods (2.60–2.95 mm vs. 3.66–3.87 mm median error; p < 0.05). No significant differences were found between SSM reconstructions from bony landmarks and SSM reconstructions from digitised landmarks obtained in the motion lab and therefore reconstructions using skin landmarks are as accurate as reconstructions from landmarks obtained from medical images.

**Significance:**

These results indicate that SSM reconstructions can be used to increase the accuracy in obtaining bone shapes from surface digitised experimental data acquired in motion lab environments.

## Highlights

• Improved scaling of bone shapes from digitised external landmarks for gait analysis.
• Scaling of articulated bones.
• Quantification of soft tissue artefact in digitisation at landmark locations.

## 1 Introduction

Knowledge of the shapes of the underlying skeletal anatomy is important for the accurate estimation of joint kinematics, kinetics and the prediction of muscle forces. Together with passive restraining structures, such as ligaments, and loading experienced, bone shapes determine the joint’s articular kinematics, including rotation axes and ranges of motion. Optical marker-based motion capture enables the quantification of kinematics and, in combination with inverse dynamics and optimisation approaches to determine muscle forces, facilitates the estimation of joint torques and joint contact forces. These calculations are sensitive to estimations of joint centres. Most commonly, medical imaging is not acquired for motion analysis and, therefore, most gait models, like the widely used Plug-in-Gait [1], rely on optical marker positions and regression equations to calculate those joint centres.

Muscle forces, joint forces and moments are of particular interest in the analysis of musculoskeletal conditions; muscle force predictions using inverse dynamics with optimisation methods are based on intersegmental forces and moments derived from kinetic analyses. Recent studies have shown that the accuracy of predicted muscle forces is sensitive to the level of subject-specificity of the anatomy [[2], [3], [4], [5], [6], [7]]. The methods to adapt a model to a subject-specific geometry include the use of bone shapes to specify muscle and ligament attachment sites [[2], [3], [4], [5]], definition of joint contact points [7] and utilising bone shapes to improve upon measured kinematics, allowing them to achieve more realistic joint motion of the knee [3] and hip [6].

The gold standard for obtaining subject-specific bone morphology is the segmentation of shapes from three-dimensional medical imaging. As these methods are too expensive and segmentations are too time-consuming to incorporate into gait analysis trials, the most commonly used method for creating subject-specific geometries is linear scaling of readily available bone geometries. These can be taken from commercial databases or published datasets [[8], [9], [10], [11]]. Parameters for linear scaling methods can be estimated from marker data of either static [12,13] or dynamic trials [14,15]. The estimation of joint centres, including the hip joint centre (HJC), and the prediction of muscle paths therefore typically relies on the digitisation of bony landmarks and the linear scaling of a reference anatomy in the Euclidian space [12,15,16]. However, anatomy varies in a non-linear way [17], and so the most commonly used scaling methods only capture anatomical variations with a limited accuracy.

Statistical shape models (SSMs) enable compact representations of non-linear variations in the Euclidian space corresponding to their probabilities within a training set. These models have been widely used for segmentation [18], characterisation [19] and shape reconstruction [20]. SSMs have been used to reconstruct shapes from sparse point data [21,22], but there are no published studies incorporating estimations of soft tissue thickness to improve the accuracy. This was addressed in the present study.

The aim of this study was to test the hypothesis that lower limb bone shape predictions from skin-based measurements, utilising an underlying SSM that corrects for soft tissue artefacts in digitisation, are more accurate than conventional linear scaling of bone geometries. The study design was to: 1) calculate relationships between soft tissue digitisation artefacts at commonly used bony landmarks estimated from landmark digitisations in magnetic resonance (MR) scans and anthropometric parameters; 2) develop a method to reconstruct the femur and tibia/fibula shapes from digitisation of landmarks obtained in the motion lab and the use of a combined SSM of the bones of the thigh and shank; and 3) reconstruct shapes using linear scaling in the Euclidian domain, comparing the accuracy of the reconstructions of linear scaling and shape models using difference measures between the surfaces, errors in predicted HJCs and anatomical measures of the tibia and femur.

## 2 Material and methods

### 2.1 Participants

The study was approved by the NHS Research Ethics Committee and written informed consent was provided according to the Committee’s guidelines. The lower limbs of 35 healthy participants (13 female, 22 male; height 155 cm–193 cm; mass 45 kg–108 kg; body mass index17.0 kg/m^2^ to 34.8 kg/m^2^; aged 23–70 years [23]) were scanned in a supine position using a 3.0 T MR scanner (MAGNETROM Verio, Siemens, Germany) using a T1 weighted axial spin echo scan (repetition time: 11.6 ms; echo time: =4.28 ms; resolution: 1.4 mm × 1.4 mm x 1.0 mm). Femur and tibia/fibula bone geometries were segmented from the MR images using a semiautomatic procedure in Mimics (v17.0, Materialise, Belgium).

**Fig. 1:**
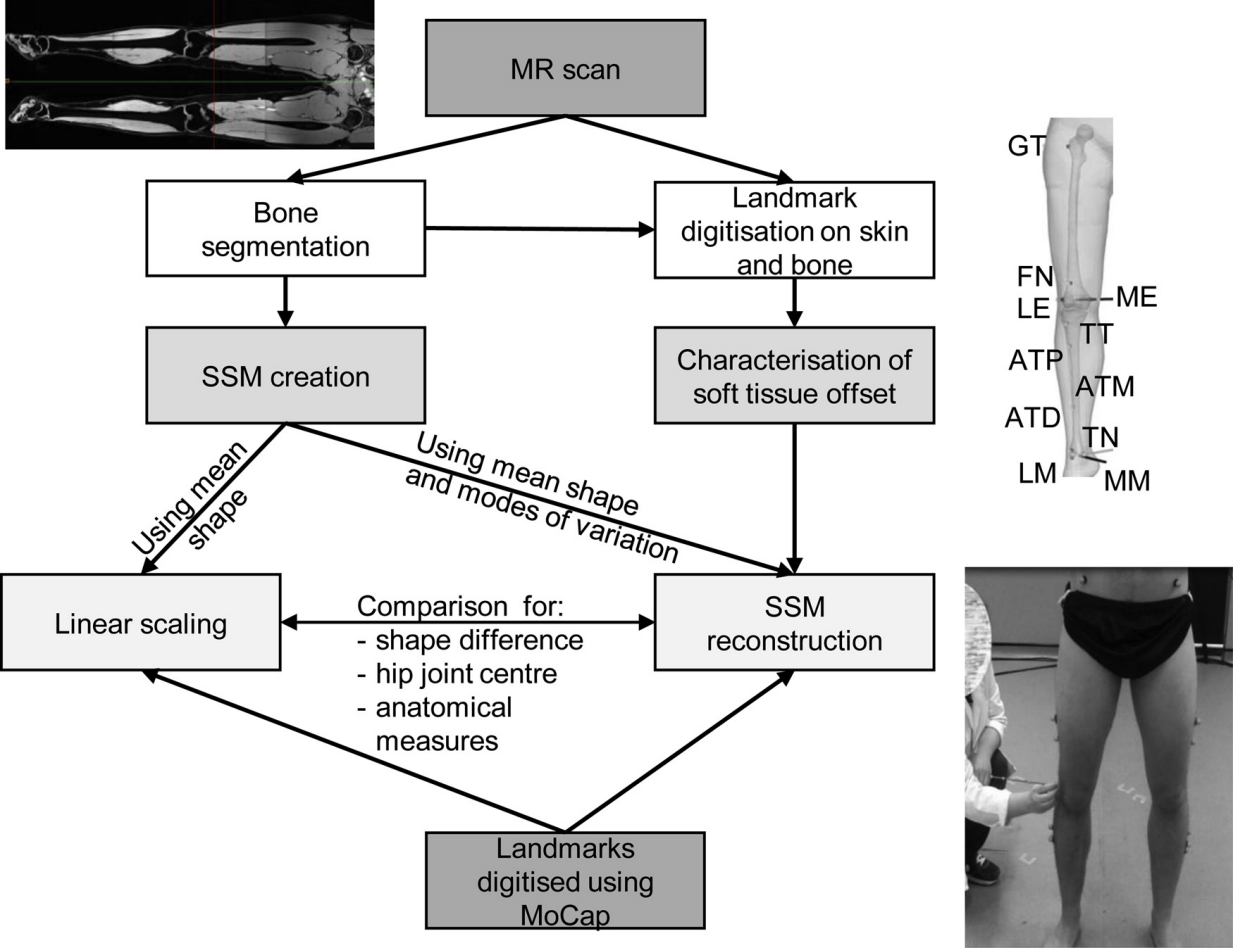
Schematic representation of the study design: Bony landmarks digitised in magnetic resonance (MR) scans on skin and bone surfaces were used to estimate soft tissue offsets. Statistical shape models were created using bone shapes segmented from MR scans. Shapes were reconstructed from shape model reconstructions and by linear scaling methods using landmark positions digitised in the motion lab. The reconstructed shapes were compared to segmented bone shapes.

For seventeen of the scanned participants, landmarks on the pelvis, thigh and shank of the left and right leg were digitised using a ten camera optical motion capture system (Vicon Ltd., UK). Digitisations were performed twice, on two separate occasions with at least a week between them. One experienced operator digitised four points on the pelvis – the left and right anterior and posterior iliac spines (LASIS, RASIS, LPSIS, RPSIS); four points on the femur – the greater trochanter (GT), the medial and lateral epicondyles (ME, LE) and the femoral notch located above the patella (FN); and seven points on the shank – the tibial tuberosity (TT), lateral and medial malleoli (LM, MM), tibial notch (TN) anterior to the middle of the ankle joint on the distal tibia, and proximal, middle and distal points along the tibial crest (ATP, ATM, ATD; Fig. 1). For registration of the measured points, clusters of three-marker were attached to the pelvis [24], right thigh and shank [25]. During the measurements, landmark positions were calculated in the cluster coordinate frames. Femoral head centre (FHC) was reconstructed using the landmark positions and the most commonly used regression equation in the literature [26]:

$$FHC=\;\left(\begin{array}{c} -0.24\;PD-9.9\\ -0.30\;PW-10.9\\ 0.33\;PW+7.3 \end{array}\right)$$

where pelvic depth (PD) was the distance between the connecting lines of ASIS and PSIS and pelvic width (PW) was the inter-ASIS distance. The radius of the femoral head (FH) was approximated by linearly scaling the radius of the mean shape of all segmented femurs (r=21.1 mm) with the ratio of the participant’s femur length to the mean femur length. Using the radius and the FHC, points superior (FHS), medial (FHM), anterior (FHA), and posterior (FHP) to the FHC relative to the local coordinate system were calculated, in addition to the digitised landmarks. Local coordinate systems were constructed using the directions from the midpoint of the LE and ME to the FHC and the midpoint of the LM and MM to the TT as y-axes for the femur and tibia, respectively. The x-axes were constructed using the component which was orthogonal to the y-axes of the connection from ME to LE and MM to LM, respectively. The z-axes were defined orthogonal to x and y-axes. To evaluate the accuracy of the scaling methods, points corresponding to the digitised points measured in the motion lab were selected on the segmented bone surfaces of all participants (Fig. 1).

### 2.2 Estimation of soft tissue artefact in digitisation

To characterise the soft tissue digitisation artefact, landmarks on the skin and on the bone surfaces were manually digitised from the MRIs using the segmented bone surfaces and their positions were corrected using the axial image slices. Soft tissue artefacts were calculated as the Euclidean distance between skin- and bone-based landmarks. Linear regression models of the artefacts were calculated for each landmark location using height, mass, age, gender and BMI. Models with an adjusted coefficient of determination larger than 0.5 were reduced by subsequently removing not relevant factors, determined by evaluating the Akaike information criterion (AIC) using R (R3.2.1, www.r-project.org). Statistical significance of the factors was tested by performing an analysis of variance with a significance level of α = 0.05 and coefficients that were not significant were removed. The resulting regression equations are listed in Table 1.

**Table 1:** Mean and standard deviations of the distances between the landmarks on the thigh and shank virtually digitised on the skin and bone surface from MR scans. Regression equations using a subset of the factors: age, height, body mass, BMI and gender (0=male, 1=female). Regression equations with an R^2^ value below 0.5 are neglected and are not shown in the table.

Segment	Landmark	Distance (mm)		R^2^ of full model	Regression
		*Mean*	*SD*		
Thigh	GT	41.99	16.72	0.61	16.2 mm - 32.01 mm * gender + 0.65 mm/kg *body mass
Thigh	FN	27.73	3.95	0.33	
Thigh	LE	15.76	4.49	0.54	2.7 mm -5.83 mm * gender + 0.71 mm*m^2^/kg * BMI
Thigh	ME	18.60	6.96	0.71	2.89 mm - 14.11 mm * gender + 0.35 mm/kg * body mass
Shank	TT	7.74	2.99	0.13	
Shank	TC1	6.54	2.14	0.39	
Shank	TC2	5.95	1.74	0.40	
Shank	TC3	7.02	2.49	0.33	
Shank	TN	14.35	2.40	0.23	
Shank	LM	4.81	0.98	0.26	
Shank	MM	5.49	0.90	0.17	

### 2.3 Shape models

Combined SSMs of the femur and tibia/fibula were generated from right and mirrored left limbs of the 35 participants. The dataset was used to create SSMs in previous studies and was shown to represent missing shapes in a leave-one-out model with accuracies of approximately 2 mm [23]. The bones were aligned to minimise the root mean square error (RMSE) between surfaces using rigid body transformations. To create point correspondences, all surfaces were registered to a reference surface using non-linear free-form deformations (IRTK [27,28],) in two steps: a deformation between selected landmarks and pseudo-landmarks and a transformation between all surface vertices using the landmark deformation as a pre-transformation. For the shape models, point correspondences for the tibia/fibula were created for each part of the model individually, without changing their relative positions. Modes of variations were calculated using a principal component analysis on the coordinate vectors of all surface vertices using a leave-one-out strategy, leaving out the shapes of the bones that were reconstructed.

### 2.4 Bone reconstruction

Shapes of the femur and tibia/fibula were reconstructed from landmarks digitised on the bone and skin surface of the MRIs and digitised in the motion lab.

#### 2.4.1 Shape model reconstruction

Bone shapes were reconstructed by morphing the SSMs to the landmarks by simultaneously optimising rigid-body transformation and shape model parameters. The optimisation used different objective functions for landmarks measured on skin or bone: for the reconstruction from skin-surface landmarks, the negative log-likelihood function of a Gaussian distribution $D=\sum_{i}0.5\text{log}\sigma_{i}^{2}+\frac{{\left(d_{i}-\;d_{i,est}\right)}^{2}}{{2\;\sigma_{i}\;}^{2}}\;$ was minimised; and for the reconstruction from bone surface landmarks, the distance between the surface point and landmark $D=\;\sum_{i}d_{i}^{2}$ was minimised. In both cases, the distances $d_{i}=\left\|x_{i}-s_{i}\right\|$ with the landmark position $s_{i}$ and the shape model surface point ${\;x}_{i}=A\;p_{i}(\lambda )+r$ were dependent on the location r and rotation A as well as the shape model parameters $\lambda =(\lambda_{1},\ldots ,\;\lambda_{m})$ describing the surface point of the shape model, $p_{i}(\lambda )$. The calculation of the log-likelihood function used the estimated soft tissue artefact at the landmark locations, $d_{i,est}$, calculated from the regression equations or using the estimated mean offsets, and the estimated standard deviation $\sigma_{i}$. The surface points $x_{i}$ corresponding to the landmarks were either fixed points defined on the surface of the SSM or, if these did not correspond to a bony landmark (in case of ATP, ATM, ATS), were determined with a closest point search. The surface and landmarks were matched by minimising the objective functions for orientation and shape model parameters simultaneously. In both cases the objective functions included a Mahalanobis distance term for the shape model parameters to penalise shapes far from the mean. The optimisation was performed using a BFGS algorithm for bound optimisation in Python (L-BFGS-B, SciPy 0.19.1, www.scipy.org) to incorporate constraints for the shape model parameters to ±3 standard deviations. The soft tissue correction method was tested by comparing reconstructions from anatomical landmarks on the bone surfaces without soft tissue corrections to reconstruction from anatomical landmarks digitised on the skin’s surface in the MR scans using soft tissue corrections.

#### 2.4.2 Linear scaling models

Shapes were also reconstructed by linearly scaling the SSM mean shapes in the Euclidian space. The scaling factors for uniform scaling were determined as the ratio of the distances between landmarks, which were the distance between the femoral head centre and the midpoint between lateral and medial epicondyles for the femur, and the distance between the tibial tuberosity and the midpoint between lateral and medial malleoli for the tibia. In the case of the landmarks obtained from bone surfaces, the femoral head centre was calculated by fitting a sphere through four points on the femoral head. In addition to uniform scaling, a scaling method using the pelvis width to scale the bone shapes in the sagittal and coronal planes was used [2,13].

### 2.5 Anatomical measures

Anatomical measures for the femoral version, angle between anatomical and mechanical axes, physio-epicondylar angle, bow angle, anatomical posterior tibial angle, mechanical medial proximal tibial angle and tibial twist were calculated using custom made scripts fitting axes to the bone surfaces [29]. The HJC locations of the reconstructed surfaces were compared to the location of the HJCs of the segmented surfaces in local coordinate frames.

### 2.6 Statistical analysis

The SSMs were analysed for generality, compactness and specificity [30]; details can be found in the supplementary data.

SSM reconstructions from anatomical landmarks segmented on the bone surface were compared to reconstructions from anatomical landmarks measured on the skin using SSM reconstruction with and without soft tissue corrections, as well as from linear scaling methods. Surfaces were compared by aligning the reconstructed and segmented surfaces using an iterative closest point algorithm and calculating the root-mean-squared deviation between them. Differences in surface distance and anatomical measures were evaluated using Kruskal-Wallace tests. If differences were found, Wilcoxon signed-rank tests with Holm corrections were used to find differences between the reconstructions. All statistical tests used a significance level of α=0.05 and were performed using R.

## 3 Results

Characterisations of soft tissue artefacts with linear regressions using gender, weight and BMI were statistically significant for the landmarks GT, LE and ME. For all other landmarks linear regression representations were not significant (Table 1).

The comparison of SSM reconstructions from the MR scans with and without soft tissue corrections for anatomical landmarks segmented on the skin and bone surfaces, respectively, did not show statistical significant differences for the femur or tibia/fibula. The median RMSEs of the reconstructed surfaces from landmarks digitised on the bone and skin surfaces were 2.66 mm compared to 2.60 mm for the femur and 2.88 mm compared to 2.90 mm for the tibia; the errors of the HJC locations were 13.82 mm compared to 16.10 mm.

**Fig. 2:**
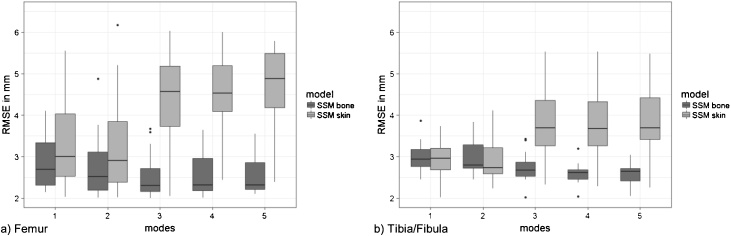
Comparison of root mean squared errors (RMSEs) of the surfaces for statistical shape model reconstructions of (a) the femur and (b) the tibia/fibula using 1 through 5 modes of variation for reconstructions from landmarks segmented on the bone surface (SSM bone) and landmarks measured on the skin surface using corrections for soft tissue artefacts (SSM skin).

RMSEs of the surface for 1 through 5 cumulative modes of variation of reconstructions with and without soft tissue corrections are shown in Fig. 2. For the reconstruction from skin landmarks (measured or segmented), reconstruction errors using 1 and 2 modes of variation were significantly smaller than reconstructions using 3, 4 or 5 modes of variation (p < 0.01). Differences between the first two modes of variation were not statistically different. Therefore the following measures were only reported for reconstructions with 1 mode of variation, which produced the smallest RMSE.

**Fig. 3:**
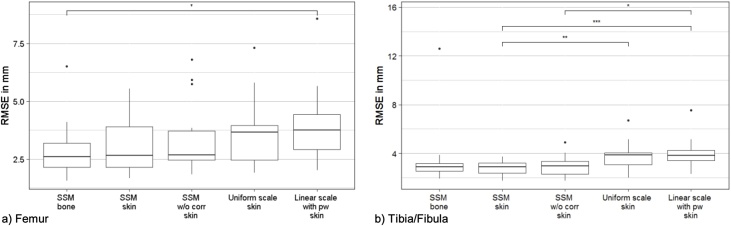
Comparison of root mean squared errors (RMSEs) of the surfaces for reconstruction of (a) the femur and (b) the tibia/fibula for statistical shape model (SSM) reconstruction from landmarks segmented on the bone (SSM bone), SSM reconstructions with (SSM skin) and without (SSM w/o corr skin) soft tissue corrections for landmarks measured on the skin, and reconstructions from landmarks measured on the skin using uniform linear scaling (Uniform scale skin) and scaling using segment length and pelvis width (Linear scale with pw skin). Differences are marked using *(p < 0.05), ** (p < 0.01) and *** (p < 0.001).

Median RMSEs of the surfaces and distances between HJCs of the tested reconstruction methods are listed in Table 2; significance levels are shown in Fig. 3. Linear scaling using the pelvis width did not show significant differences when compared to uniform scaling, but was significantly worse than SSM reconstructions from bony landmarks (femur) and SSM reconstructions from skin landmarks (tibia/fibula). No statistically significant differences were found between the SSM reconstructions from bony landmarks using a distance minimisation and reconstructions from measured skin landmarks using the correction method.

**Table 2:** Median and interquartile ranges of surface root mean squared errors and distances between segmented and reconstructed hip joint centre locations for reconstructions of the femur and tibia/fibula from a statistical shape model (SSM) using one mode of variation, uniform scaling and scaling using segment length and pelvis width. For a significance level of α=0.05, significant differences between values are indicated with identical superscripts.

Reconstruction method	Landmark set	Tibia/Fibula (mm)	Femur (mm)	Hip joint centre distance (mm)
*SSM reconstruction*	*Segmented bone*	2.88 (0.62)	2.60^a^ (1.05)	13.82*^,†^ (9.62)
	*Measured skin*	2.95^A^ (1.03)	2.68 (1.26)	17.02 (14.07)
*SSM reconstruction with soft tissue correction*	*Measured skin*	2.90^B^ (0.82)	2.66^b^ (1.74)	16.10 (10.61)
*Uniform scaling with segment length*	*Measured skin*	3.87 (0.96)	3.66 (1.50)	22.07* (8.71)
*Scaling with segment length and pelvis width*	*Measured skin*	3.84^A, B^ (0.82)	3.76^a,b^ (1.51)	22.40^†^ (9.54)

A, a, b, *, †: p < 0.05; B: p < 0.01.

**Fig. 4:**
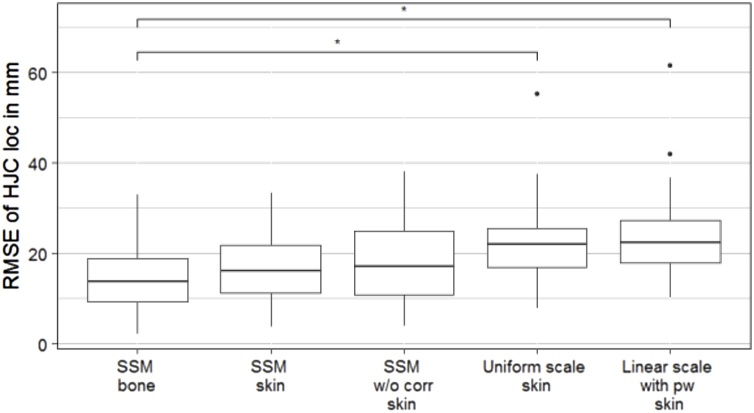
Comparison of root mean squared errors (RMSEs) in the location of the hip joint centre (HJC) locations in reconstructions from statistical shape models using digitised bone (SSM bone) and skin landmarks with (SSM skin) and without (SSM w/o corr skin) correction for soft tissue artefacts, and reconstructions from landmarks measured on the skin using uniform linear scaling (Uniform scale skin) and scaling using segment length and pelvis width (Linear scale with pw skin). Differences are marked using *(p < 0.05).

Errors in HJCs did not show statistically significant differences between shape model reconstructions from segmented bone and skin surface landmarks. Average errors in the linearly scaled models were larger than errors from shape model reconstructions, but the differences were only statistically significant for reconstructions from segmented bony landmarks (Fig. 4). There were no statistically significant differences found in any other anatomical measure; detailed results are in the supplementary data.

## 4 Discussion

This study compared linear scaling and SSM-based reconstruction methods for lower limb bones using sparse landmarks obtained by digitisation on the skin surface. Soft tissue digitisation artefacts were characterised using measurements from MR images and a method for the reconstruction of SSMs, correcting the measured landmark positions, was tested.

The description of soft tissue artefacts in this study showed a moderate correlation between BMI, body mass and gender for landmarks at the epicondyles and the greater trochanter. For these landmarks, gender had a particularly large influence on the measured skin artefact. For all other measured landmarks, the standard deviations were small and regression equations did not show a significant improvement in the prediction of the skin artefact. Previous publications have reported soft tissue artefacts for the shoulder, spine and pelvis [[31], [32], [33]]; however, previous publications have not used soft tissue offset characterisations in the lower limb for bone shape reconstructions. Limitations of the presented methodology are differences in the position for characterising and applying soft tissue offsets, i.e. a supine position in the MR scan versus an upright position in the motion laboratory, which could be overcome if an upright MR scanner would be available; unavoidable soft tissue compressions by the rater during digitisation, which might have influenced the measurements; and a limited number of available subjects used to create the statistical shape model, which could be increased in future studies. Further, variations in the joint centre estimation due to cartilage thickness was not addressed in this study. Accuracy improvements to this type of study could potentially be made by the use of other medical imaging modalities, such as CT scanning which is not advocated due to the effects of ionising radiation.

Shape model reconstructions had, on average, more accurate surface predictions than when using linear scaling methods and were comparable to those reported by Zhang et al. [22]. There were no differences between SSM reconstructions from segmented bony landmarks alone and SSM reconstructions from segmented and measured skin landmarks. Reconstructions from skin landmarks were most accurate for one and two modes of variation. This was most likely caused by the small number of landmarks used for reconstruction and the soft tissue artefacts and corrections, which might cause an overfitting of the model to the landmarks given the uncertainty from the soft tissue artefacts.

Knowledge of the position of the HJC is required in musculoskeletal modelling to specify the moment arms of muscles around the joint. The errors in the HJC locations of the reconstructions from bony landmarks (13.8 mm) and skin landmarks using soft tissue corrections (16.1 mm) were within the range reported by previous studies [26,34,35].

A limitation of the comparison of HJC errors is the use of measured landmarks, especially the greater trochanter, for the creation of a local coordinate system. Since this landmark position has the largest soft tissue digitisation artefact of all measured landmarks, the local coordinate frame mixes measurement errors with inaccuracies of the landmark measurements.

Gait analysis is often performed for paediatric patients and the method shown here has potential for this population. However, specifically created SSMs that represent the studied population are required and since the bone shapes in children can have large variations it might require specific SSMs to represent specific developmental stages.

Although this study sought to provide reconstructions for rigid-body musculoskeletal simulations, other potential applications could be considered, such as computational biomechanics using finite element analysis to simulate arthrokinematics and tissue stresses and strains. The accuracy found here would potentially enable this. To evaluate the impact on musculoskeletal simulations, further analysis with specific simulations is required.

In conclusion, it has been shown in this study that bone shape reconstructions using landmarks measured in a motion lab can be improved using SSMs when compared to linear scaling algorithms in an adult population. Therefore, this technology could now be used to create subject-specific musculoskeletal models without the need for additional medical imaging.

## CRediT authorship contribution statement

**Daniel Nolte:** Conceptualization, Methodology, Software, Formal analysis, Writing - original draft. **Siu-Teing Ko:** Investigation, Data curation, Writing - review & editing. **Anthony M.J. Bull:** Conceptualization, Methodology, Formal analysis, Supervision, Writing - review & editing, Funding acquisition. **Angela E. Kedgley:** Conceptualization, Methodology, Investigation, Formal analysis, Data curation, Supervision, Writing - review & editing.

## Declaration of Competing Interest

The authors (Daniel Nolte, Siu-Teing Ko, Anthony M.J. Bull and Angela E. Kedgley) do not have any financial or personal relationships with other people or organisation that could inappropriately influence their work.
