# Supplementary material for: Reconstruction of the lower limb bones from digitised anatomical landmarks using statistical shape modelling
Source: Gait Posture. 2020 Mar;77:269–75. doi: 10.1016/j.gaitpost.2020.02.010 (PMC7090904; doi:10.1016/j.gaitpost.2020.02.010)
Supplement: Supplementary file 1 [file mmc1.docx]

**Reconstruction of the lower limb bones from digitised anatomical landmarks using statistical shape modelling – Supplementary data**

**Daniel Nolte, Siu-Teing Ko, Anthony MJ Bull, Angela E Kedgley**

**Analysis of the statistical shape model**

For the analysis of the statistical shape models (SSMs) used in the present study, an SSM representing all shapes in the study was analysed to evaluate the compactness and specificity [1]. The SSM was evaluated for compactness, specificity and generalisability. For the analysis of the compactness, the cumulative variability was evaluated (Figure S1). The first mode of variation represents 82.87% of total variations in the model, the second 3.75% and the third 2.61%.

|  |
| --- |
| **Figure S1:** Cumulative variance represented by the first 25 modes of variation of the statistical shape model. The first mode of variation represents about 82% of total variation, consecutive modes represent less than 4% each. |

The specificity was evaluated by calculating minimum root-mean-squared deviation between randomly generated shapes using the first n modes of variation with coefficients between -3 and 3 standard deviations and the closest shape in the training set. An estimator for the specificity using 20 randomly generated shapes is presented in Figure S2.

The generality of the SSM was evaluated by reconstructing the shapes of the training set using leave-one-out statistical shape models. Shapes were reconstructed by matching all points of the surfaces of the input shapes to the closest surface points of the shape model instance, separate for femur and tibia/fibula, and calculating the root-mean-squared error. The parameters of the shape model were estimated to minimise the average root-mean-squared error using a sequential quadratic optimisation algorithm (SLSQP, SciPy 0.19.1, [www.scipy.org](http://www.scipy.org)). The average root-mean-squared errors are shown in Figure S3.

Shapes of the combined SSM representing the variances captures in the first three modes of variation are shown in Figure S4.

|  |
| --- |
| **Figure S2:** Plot of the root mean squared error (RMSE) of the closest shape in the training set to 20 shapes randomly generated by varying the first n modes of variation in order to test the specificity of the statistical shape model. |
|  |
| **Figure S3:** Plot representing the generality of the statistical shape model: Average root-mean-squared errors (RMSE) with standard deviations of shapes of the training set reconstructed from leave-one-out statistical shape models. |

| 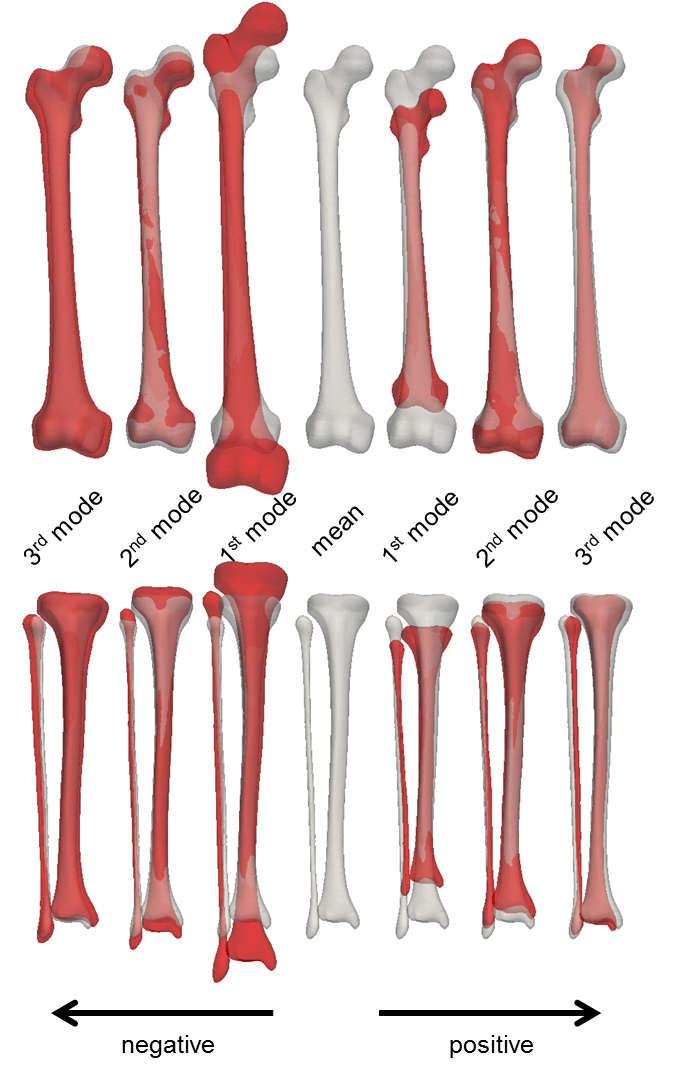 |
| --- |
| **Figure S4:** Representation of the first three modes of variation of the combined statistical shape model of femur and tibia/fibula showing the mean (middle) and shapes of ±2 standard deviations of single modes. The first mode represents the variation of size (length and width) of the bones; the second represents mainly variations in length while maintaining the width; the third mode of variation represents mainly the width without changes in length. |

**Reconstruction results**

The shapes reconstructed in this study were evaluated for accuracy in anatomical measures. The anatomical measures used in this study were described in [2]. The tibial angles were calculated using an axis through the malleoli and axis connecting the medial and lateral extreme points of the tibial plateau projected to plane fitted through the tibial plateau as medial-lateral (M/L) axis, and an axis orthogonal to the M/L axis in the same plane, and a line fitted along the tibial shaft. Tibial plateau angle in M/L and A/P directions were calculated as angle between tibial long axis and M/L and A/P tibial plateau axis, respectively. The tibial twist was calculated as angle between M/L tibial plateau axis in intra-malleoli axis, projected to the plane orthogonal to the tibia long axis.

Mean values of the measures for the segmented bone surfaces are shown in Table S1, median errors between segmented and reconstructed bones are shown in Table S2. The median directional error of the estimation of the hip joint centre is shown in Table S3.

| \| \| **Measure** \| **Segmented bones** \| \| --- \| --- \| \| **Femoral neck angle (°)** \| 50.14 (5.38) \| \| **Anat.-mech. axis angle (°)** \| 6.52 (0.88) \| \| **Version epi (°)** \| 9.16 (14.75) \| \| **Version post (°)** \| 11.92 (12.73) \| \| **Diaph.-cond. angle (°)** \| 81.90 (3.91) \| \| **Bow angle (°)** \| 0.67 (1.48) \| \| **Femoral head radius (mm)** \| 21.44 (3.17) \| \| **Tibial plateau angle M/L (°)** \| 89.13 (1.05) \| \| **Tibial plateau angle A/P (°)** \| 84.92 (1.13) \| \| **Tibia twist** \| 17.71 (9.53) \| \| \| --- \| --- \| --- \| --- \| --- \| --- \| --- \| --- \| --- \| --- \| --- \| --- \| --- \| --- \| --- \| --- \| --- \| --- \| --- \| --- \| --- \| --- \| --- \| \| **Table S1:** Median values and interquartile range (in brackets) of anatomical measures for segmented bones used as training set for the statistical shape model. \| |
| --- | --- | --- | --- | --- | --- | --- | --- | --- | --- | --- | --- | --- | --- | --- | --- | --- | --- | --- | --- | --- | --- | --- | --- | --- |
|  |
| \| **Reconstruction method** \| *SSM reconstruction* \| \| *SSM reconst. with soft tissue correction* \| *Uniform scaling with segment length* \| *Scaling with segment length and pelvis width* \| \| --- \| --- \| --- \| --- \| --- \| --- \| \| **Landmark set** \| *Segmented bone* \| *Measured skin* \| *Measured skin* \| *Measured skin* \| *Measured skin* \| \| **Femoral neck angle (°)** \| 3.49 (2.40) \| 3.30 (2.77) \| 3.30 (2.77) \| 3.49 (2.22) \| 2.96 (2.81) \| \| **Anat.-mech. axis angle (°)** \| 0.67 (0.49) \| 0.64 (0.52) \| 0.64 (0.52) \| 0.67 (0.46) \| 0.60 (1.09) \| \| **Version epi (°)** \| 5.49 (8.89) \| 5.57 (9.38) \| 5.57 (9.38) \| 5.49 (8.81) \| 6.83 (8.80) \| \| **Version post (°)** \| 5.95 (7.04) \| 6.10 (7.20) \| 6.10 (7.20) \| 5.95 (7.21) \| 6.58 (7.21) \| \| **Diaph.-cond. angle (°)** \| 1.53 (1.94) \| 1.48 (1.87) \| 1.48 (1.87) \| 1.53 (1.90) \| 1.46 (2.12) \| \| **Bow angle (°)** \| 0.86 (0.87) \| 0.68 (0.74) \| 0.68 (0.74) \| 0.86 (0.98) \| 0.72 (0.97) \| \| **Femoral head radius (mm)** \| 0.74 (1.48) \| 0.77 (1.97) \| 0.77 (1.97) \| 0.74 (1.36) \| 1.18 (1.39) \| \| **Tibial plateau angle M/L (°)** \| 0.59 (0.71) \| 0.53 (0.69) \| 0.53 (0.69) \| 0.59 (0.64) \| 0.49 (0.68) \| \| **Tibial plateau angle A/P (°)** \| 1.90 (2.17) \| 1.79 (2.00) \| 1.79 (2.00) \| 1.90 (1.82) \| 3.89 (1.69) \| \| **Tibia twist** \| 6.01 (11.45) \| 6.79 (10.77) \| 6.79 (10.77) \| 6.01 (9.76) \| 9.41 (9.75) \| |
| **Table S2:** Median errors (with interquartile ranges) in anatomical measures for surface reconstructions compared the surfaces segmented from medical images. The measures include the femoral neck angle, angle between anatomical and mechanical axis of the femur, femoral version angle using the axis calculated the posterior peaks of the condyles (PCA) and the trans-epicondylar axis (TEA), diaphyseal-condylar angle, femoral bow angle, femoral head radius, tibial plateau angle in anterior-posterior (A/P) and medial-lateral (M/L) direction, and tibial twist. |

| \| **Reconstruction method** \| **Landmark set** \| *X (mm)* \| *Y (mm)* \| *Z (mm)* \| \| --- \| --- \| --- \| --- \| --- \| \| *SSM reconstruction* \| *Segmented bone* \| 5.09 (6.74) \| 5.55 (5.40) \| 7.22 (14.86) \| \| *Measured skin* \| 4.45 (7.35) \| 5.97 (5.59) \| 6.90 (14.91) \| \| *SSM reconstruction with soft tissue correction* \| *Measured skin* \| 4.43 (6.20) \| 8.30 (10.01) \| 7.51 (13.79) \| \| *Uniform scaling with segment length* \| *Measured skin* \| 6.24 (6.81) \| 14.06 (15.38) \| 7.85 (13.39) \| \| *Scaling with segment length and pelvis width* \| *Measured skin* \| 5.32 (5.84) \| 14.49 (16.48) \| 8.52 (13.66) \| |
| --- | --- | --- | --- | --- | --- | --- | --- | --- | --- | --- | --- | --- | --- | --- | --- | --- | --- | --- | --- | --- | --- | --- | --- | --- | --- | --- | --- | --- | --- |
| **Table S3:** Median direction errors with interquartile ranges of the hip joint centre of reconstructions compared to hip joint centre location of the bone shapes segmented form medical images in the local reference frame. Statistical shape model (SSM) reconstructions were calculated using only the first mode of variation. |

Direct comparison of the reconstruction errors for each subject of each individual showed that in more than 59% of the cases for the femur and more than 76% for the tibia/fibula, the RMSE errors of the shape model reconstructions were smaller than reconstructions using one of the linear scaling methods (Table S4).

| \| ***Femur*** \| *Uniform scaling with segment length* \| *Scaling with segment length and pelvis width* \| \| --- \| --- \| --- \| \| *SSM reconstruction. with soft tissue correction* \| 59% \| 82% \| \| *SSM reconstruction* \| 71% \| 76% \|  \| ***Tibia/fibula*** \| *Uniform scaling with segment length* \| *Scaling with segment length and pelvis width* \| \| --- \| --- \| --- \| \| *SSM reconstruction. with soft tissue correction* \| 88% \| 82% \| \| *SSM reconstruction* \| 76% \| 76% \| |
| --- | --- | --- | --- | --- | --- | --- | --- | --- | --- | --- | --- | --- | --- | --- | --- | --- | --- | --- |
| **Table S4:** Frequencies of cases in which SSM reconstructions using one mode of variation had lower errors than linear scaling methods. |

**References**

[1] Davies R, Twining C, Taylor C. Statistical Models of Shape. vol. 25. 2008. doi:10.1007/978-1-84800-138-1.

[2] Nolte D, Bull AM. Femur finite element model instantiation from partial anatomies using statistical shape and appearance models. Med Eng Phys 2019. doi:10.1016/j.medengphy.2019.03.007.
